# *In Vitro* Sensitivity to Venetoclax and Microenvironment Protection in Hairy Cell Leukemia

**DOI:** 10.3389/fonc.2021.598319

**Published:** 2021-07-26

**Authors:** Alexia Vereertbrugghen, Ana Colado, Ernesto Gargiulo, Raimundo Fernando Bezares, Horacio Fernández Grecco, Gregorio Cordini, Maria del Rosario Custidiano, Jean-Hugues François, Guy Berchem, Mercedes Borge, Jerome Paggetti, Etienne Moussay, Romina Gamberale, Mirta Giordano, Pablo Elías Morande

**Affiliations:** ^1^ Instituto de Medicina Experimental (IMEX)–CONICET–Academia Nacional de Medicina (ANM), Buenos Aires, Argentina; ^2^ Tumor Stroma Interactions, Department of Oncology, Luxembourg Institute of Health, Luxembourg, Luxembourg; ^3^ Sección Hematología, Hospital General de Agudos Dr. Teodoro Álvarez, Buenos Aires, Argentina; ^4^ Sección Hematología, Sanatorio Municipal Dr. Julio Méndez, Buenos Aires, Argentina; ^5^ Hospital de Clínicas José de San Martín, Universidad de Buenos Aires, Buenos Aires, Argentina; ^6^ Servicio de Hematología y Transplante de Médula Ósea, Instituto Alexander Fleming, Buenos Aires, Argentina; ^7^ Laboratory of Hematology, Centre Hospitalier de Luxembourg, Luxembourg, Luxembourg; ^8^ Department of Hemato-Oncology, Centre Hospitalier de Luxembourg, Luxembourg, Luxembourg; ^9^ Departamento de Microbiología, Parasitología e Inmunología, Facultad de Medicina, Universidad de Buenos Aires, Buenos Aires, Argentina

**Keywords:** hairy cell leukemia, ABT-199, cell death, leukemia microenvironment, venetoclax

## Abstract

Current standard treatment of patients with hairy cell leukemia (HCL), a chronic B-cell neoplasia of low incidence that affects the elderly, is based on the administration of purine analogs such as cladribine. This chemotherapy approach shows satisfactory responses, but the disease relapses, often repeatedly. Venetoclax (ABT-199) is a Bcl-2 inhibitor currently approved for the treatment of chronic lymphocytic leukemia (CLL) and acute myeloid leukemia (AML) in adult patients ineligible for intensive chemotherapy. Given that HCL cells express Bcl-2, our aim was to evaluate venetoclax as a potential therapy for HCL. We found that clinically relevant concentrations of venetoclax (0.1 and 1 µM) induced primary HCL cell apoptosis *in vitro* as measured by flow cytometry using Annexin V staining. As microenvironment induces resistance to venetoclax in CLL, we also evaluated its effect in HCL by testing the following stimuli: activated T lymphocytes, stromal cells, TLR-9 agonist CpG, and TLR-2 agonist PAM3. We found decreased levels of venetoclax-induced cytotoxicity in HCL cells exposed for 48 h to any of these stimuli, suggesting that leukemic B cells from HCL patients are sensitive to venetoclax, but this sensitivity can be overcome by signals from the microenvironment. We propose that the combination of venetoclax with drugs that target the microenvironment might improve its efficacy in HCL.

## Introduction

Hairy cell leukemia (HCL) is an incurable lymphoproliferative B cell malignancy of low incidence characterized by the presence of pancytopenia, splenomegaly, and infiltration of leukemic cells in the bone marrow, spleen, and liver (1, 2). Current standard treatment with the purine analog cladribine shows a partial good response, but patients relapse repeatedly and develop refractoriness that raises up to 40% of cases (3). Novel therapy strategies being presently tested include immune toxin-based targeting of tumor cells and kinase inhibitors, among others (4–6).

HCL cells express B cell lymphoma 2 (Bcl-2) (7, 8), an anti-apoptotic protein that plays a central role in evading programed cell death, promoting tumor growth and disease progression in cancer (9). A rising number of clinical trials based on Bcl-2 inhibition are currently ongoing for different hematological malignancies and also for solid cancers (10). The BH3 mimetic venetoclax (ABT-199) is a potent Bcl-2 selective inhibitor (11) approved for the use in chemotherapy-unfit patients with chronic lymphocytic leukemia (CLL) and older acute myeloid leukemia (AML), able to induce rapid apoptosis in blood-derived leukemic cells *in vitro* and showing promising results in the clinic (12–14). Despite this, the efficacy of drugs that selectively inhibit Bcl-2 has not been studied in HCL yet.

The tumor microenvironment exerts a protective role that withholds the levels of Bcl-2-induced cell death. In CLL, the complex cross-talk between leukemic cells and their milieu drives proliferation, disease progression, and therapy refractoriness (15). The pro-survival supportive niche localizes in the lymph nodes and bone marrow of patients, the anatomical site where CLL cells proliferate, and is composed of accessory myeloid cells, stromal cells, and T lymphocytes, among others (16). Activation of CLL cells through interactions provided by autologous T cells and TLR8 signaling accompanied by IL-2 stimulation lead to resistance to venetoclax-induced apoptosis of the leukemic clone (17, 18). In HCL, the niche interactions between tumor cells and the normal counterpart cells play a key role as well (19). HCL patients present a fibrotic and infiltrated bone marrow, and leukemic cells show high affinity to mesenchymal bone marrow stromal cells (BMSCs) in *in vitro* studies (20). The cross-talk between HCL cells and BMSCs leads to activation of signaling pathways such as mitogen activated protein (MAP) kinases and the nuclear factor *κ*B (NF-*κ*B) pathway and to inhibition of apoptosis induced by treatment with BRAF inhibitors (21). Peripheral blood T lymphocytes and also those present in the characteristic leukemia infiltrated spleen of HCL patients are expanded and can recognize autologous HCL cells (22). It has been proposed that rather than the growth control of neoplastic cells, such T-cell recognition favors HCL cell survival and thus disease progression (23).

In this work, we studied the effect of venetoclax in HCL to test its therapeutic potential. We evaluated the capacity of the drug to induce cell death in patient-derived leukemic cells from the blood and also studied its effect in non-malignant NK cells and T lymphocytes. Because the cross-talk between tumor cells and their milieu is relevant for understanding the mechanisms that lead to drug resistance, we also evaluated the effect of venetoclax-induced cell death on microenvironment-activated HCL cells.

## Materials and Methods

### Patients

The eight HCL peripheral blood samples used in this study were obtained after informed consent in accordance with the Declaration of Helsinki and with Institutional Review Board approval from the National Academy of Medicine, Buenos Aires, Argentina, and the Comité National d’Ethique de Recherche (Luxembourg). HCL was diagnosed according to standard criteria. At the time of analysis, all patients were free from clinically relevant infectious complications and had not been treated for at least two years. To obtain the peripheral blood mononuclear cells (PBMCs) from HCL patients, a density gradient centrifugation (Ficoll-Paque, GE Healthcare) was developed. PBMC samples were used fresh in cases #8, #14, #15, #18, and #23 or frozen in cases #1, #2, and #9 (in 10% DMSO, 45% FBS, and 45% RPMI) and stored in liquid nitrogen until thawed to be used.

### Primary Cultures and HS-5 Cell Line

PBMCs (2.5 × 10^5^ cells in 150 µl) from HCL patients were cultured using 96-well plates in RPMI 1640 + 10% FCS alone (control), or in the presence of PAM3 used at 300 ng/ml (Invivogen, San Diego, California, USA #tlrl-pms), CpG used at 5 μM (Invivogen, San Diego, California, USA #tlrl 2216), or immobilized anti-CD3 (*α*CD3) used at 0.5 μg/ml (Biolegend, San Diego, California, USA # 300302). After 24 h of culture, a part of HCL cells was collected to assess their activation, and another fraction was incubated with DMSO as control or with venetoclax (ABT-199, MedKoo, Morrisville, North Carolina, USA) at 0.1 or 1 µM, in all the conditions detailed above. After another 24 h of culture, HCL cells were collected to further assess activation and to evaluate cell death. HS-5 cell line was purchased from ATCC, Manassas, Virginia, USA (CRL-11882).

### Flow Cytometry Studies

For surface antigen staining, cells were incubated for 30 min at 4°C with the corresponding antibodies diluted in PBS supplemented with 0.5% BSA and washed twice in the same buffer before acquisition. Activation of HCL cells was evaluated by measuring the expression of CD69 (BD, Bergen, New Jersey, USA Biosciences #555531) at 24 h of culture, or CD86 (BD, Bergen, New Jersey, USA Biosciences #555658, Biolegend #305412 or # 374203) at 48 h of culture, and anti-CD19 (clone J3-119, Beckman Coulter, Brea, California, USA # A07771 or Biolegend #302254). For immunoglobulin light chain usage determinations, anti-Kappa PE and anti-Lambda PE antibodies were purchased from BD, Bergen, New Jersey, USA Biosciences (#555792 and 555797, respectively). Antibodies used to detect CD25, CD4, and CD56 were purchased from BD Biosciences, Bergen, New Jersey, USA (#555431, 555347 and 555517, respectively). Antibodies used for CD103 and CD8 were purchased from Biolegend (#121406 and 300908, respectively). Annexin V staining and Apotracker Green (Biolegend #427402) were used to evaluate the percentage of cell death induced by venetoclax. After the incubation with the drug, PBMCs were stained for CD19 and next incubated for 20 min at room temperature with Annexin V-FITC (Biolegend #640905) in the corresponding binding buffer to discriminate viable and dead cells. Flow cytometry analysis was performed immediately after incubation, by gating HCL cells or normal lymphocytes in the forward *versus* side scatter and by studying the CD19+ and CD19 negative populations. Cell death was corroborated by flow cytometric alterations of light-scattering properties. Cells were acquired on a FACScalibur cytometer (Becton Dickinson) or FACSARIA III (BD Biosciences). Data were analyzed with FlowJo 10.6.1 software (Tree Star, Inc).

### Statistical Analysis

Paired one-way analysis of variance (ANOVA) using correction followed by multiple comparison tests or two-tailed paired Student’s *t*-tests was performed to evaluate statistical significance. Data sets that did not pass normality tests were analyzed using Friedman test followed by Dunn’s multiple comparisons. Variables with *P* <.05 were considered to be significant. All analyses were performed using GraphPad Prism 8 software version 8.0.01 (GraphPad, San Diego, CA).

## Results

### Venetoclax Induces Cell Death in Primary HCL Leukemic Cells

**Figure 1:**
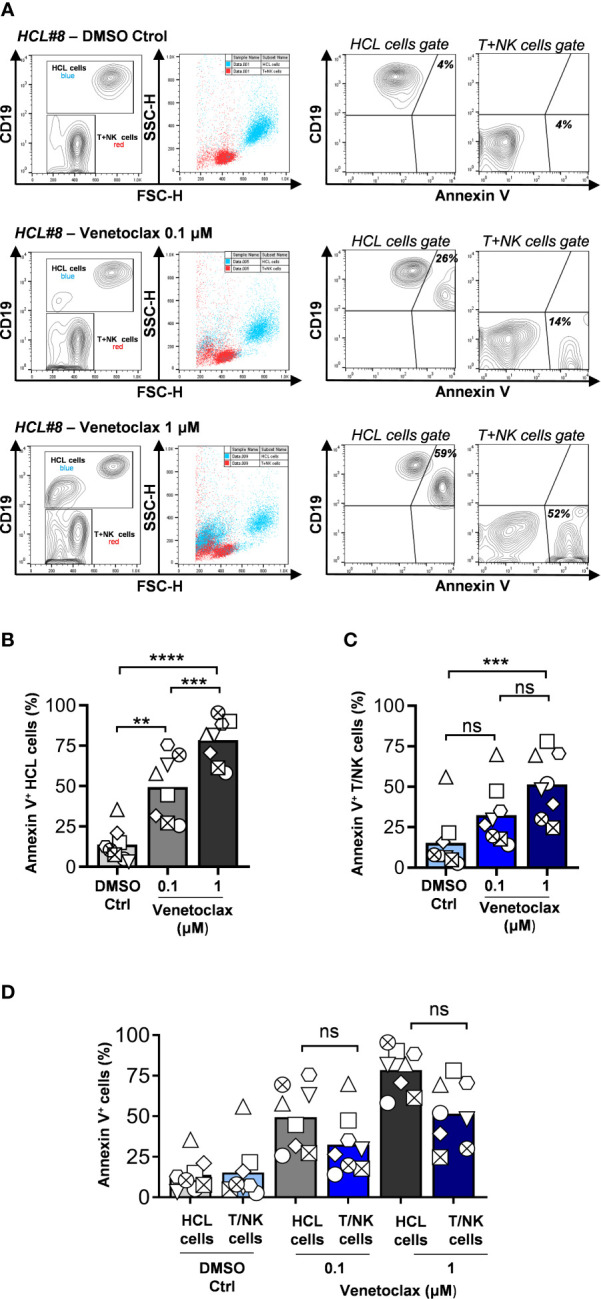
HCL cells are sensitive to venetoclax-induced cell death *in vitro*. **(A)** PBMCs were obtained by Ficoll-Paque centrifugation from peripheral blood of eight HCL patients, incubated with venetoclax for 24 h, and cell death was analyzed by flow cytometry. Leukemic cells or T + NK cells were discriminated according to CD19 expression and forward side scatter, and next gated to determine binding of Annexin V. Results from a representative sample (HCL#8) are shown. **(B)** HCL cell death induced by venetoclax for the whole cohort evaluated. **(C)** T + NK cell death induced by venetoclax for the whole cohort evaluated. **(D)** Comparison of cell death induced in HCL cells and T + NK cells. For all the group graphics, each symbol represents a single patient (n = 8, **p < 0.01, ***p < 0.001, ****p < 0.0001, paired one-way ANOVA using correction for **(B)** and Friedman test followed by Dunn’s multiple comparisons test for **(C, D)**, ns, not significant).

To test the effect of venetoclax in HCL, we developed primary cultures using PBMC samples obtained from eight diagnosed patients. Six of them belong to the classic HCL group, whereas two were variant HCL cases. Additional characteristics of the cohort enrolled in this study are detailed in **Table 1**. History of previous treatment/s and infection/s is included in **Supplementary Table S1**. Using the flow cytometry FSC and SSC parameters, HCL cells can be distinguished from normal lymphocytes based on increased size and granularity (24) (**Figure 1A** and **Supplementary Figure S1**). More than 95% of cells within this gate were CD19+ kappa+ or CD19+ lambda+, corroborating their leukemic origin. The cohort approached the expected frequency of immunoglobulin light chain gene usage in HCL of 50% (25) (**Table 1**).

**Table 1:** Characteristics of HCL patients included in this study.

Patient ID/code	Sex	Age	HCLc/HCLv	Flow cytometry phenotype	WBC count (µl)	Hematocrit (%)	Platelets (μl)	% HCL Cells in PBMC	Light chain usage	IGHV gene usage	BRAF V600E mutation
HCL#1 ◊	M	88	Variant [molecular]	CD19^+^, CD20^++/+^, CD10^−^ CD38^−^, CD5^−^, CD103^+^ LAIR^++^, CD25^+^	3,840	22	8,000	15.1	λ	IGHV 4-34	No
HCL#2 □	M	58	Variant [phenotypic]	CD45^++^, CD19^++^, CD20^++^ CD10^+^, CD38^−/dim^, CD5^−^ CD81^+^, CD43^−^, CD103^+v^ LAIR^++v^, CD11c^++v^, CD25^−^	1,570	22	21,000	34.2	λ	IGHV 2–70	ND
HCL#8 ○	M	61	Classic	CD19^+^, CD20^++/+^, CD10^+^ CD38^−^, CD5^−^, CD305^++^ CD103^++^, CD11c^++^, CD25^+^	2,500	35	53,000	28.4	λ	IGHV 5–51	ND
HCL#9 Δ	M	32	Classic	CD19^+^, CD20^+^, CD10^−^, CD38^−^, CD5^−^, CD43^−^, LAIR^++^ CD103^+^, CD11c^+^, CD25^+^	3,800	42	85,000	24.3	κ	IGHV 3–30	Yes
HCL#14 ▽	M	62	Classic	CD19^+^, CD20^+^, CD10 CD38^−^, CD5−, CD81^−^, CD305^++^, CD103^+^ CD11c^+^, CD25^+^	2,500	42	137,000	44	κ	ND	Yes
HCL#15 ⬡	M	70	Classic	CD19^++^, CD20^++^, CD103^+^ CD25^+^,LAIR 1^++^, CD11c^++^ CD45^++^, CD38^−^, CD10^+/−^ CD43^−^, CD23^−^, CD5^−^	3,000	37.3	83,000	10.3	λ	ND	Yes
HCL#18 ☒	M	46	Classic	CD19^+^, CD20^+^, CD11c^+^,CD25^+^, CD103^+^, CD123^+^	3,680	38.5	75,000	10.8	κ	ND	Yes
HCL#23 ⨂	M	58	Classic	CD19^+^, CD20^+^, CD11c^+^,CD25^+^, CD103^+^, CD123^+^	8,800	33.6	46,000	50.4	λ	ND	Yes

M, male; WBC, white blood cell count; κ, kappa immunoglobulin light chain; λ, lambda immunoglobulin light chain; IGHV, immunoglobulin heavy chain variable; ND, not determined. Symbols assigned to each patient in this table are depicted in the subsequent figures to allow tracking of particular cases.

PBMCs were exposed to clinically relevant concentrations of venetoclax (0.1 and 1 µM) for 24 h, and cell death was evaluated by Annexin V binding and flow cytometry analysis (**Figure 1A**). Venetoclax significantly augmented cell death in HCL cells at both concentrations tested in a dose-dependent manner (**Figure 1B**). Three samples were additionally evaluated at 48 and 96 h of incubation, showing increased cell death in leukemic cells that reached up to 90% of positivity (**Supplementary Figure S2**). Non-malignant lymphocytes (T + NK cells) were sensitive to venetoclax at 1 µM, but not when the drug was added at 0.1 µM (**Figure 1C**). Since it was previously reported that T cells from CLL patients and healthy donors are less sensitive to venetoclax (17, 26), we compared the levels of cell death induced in HCL cells and in T + NK cells without finding a significant differential sensitivity to this drug in our experimental settings (**Figure 1D**).

### Activation of HCL Cells Enhances Resistance to Venetoclax-Induced Cell Death

**Figure 2:**
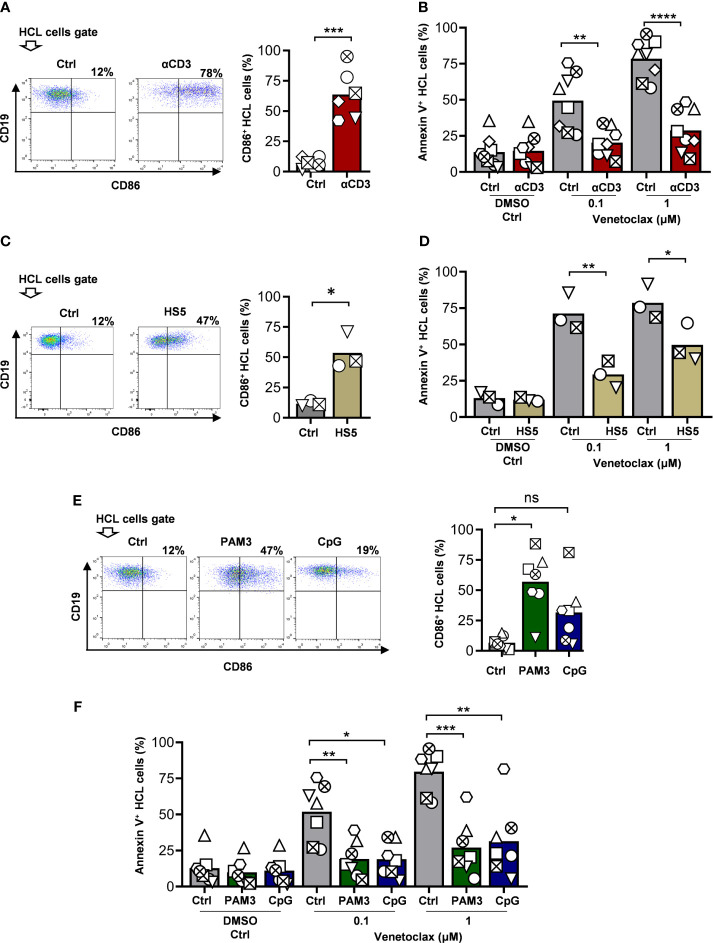
Activation of HCL cells confers resistance to venetoclax-induced cell death. **(A)** PBMCs from HCL patients were incubated with medium alone (Ctrl) or on anti-CD3-coated wells (25 ng). At 48 h, cells were labeled with anti-CD19 and anti-CD86. Shown are representative dot plots (left) and mean values (right) for n = 6 samples (two-tailed paired *t-*test). **(B)** PBMCs from HCL patients were incubated with medium alone (Ctrl) or on anti-CD3-coated wells for 24 h. Then venetoclax (0.1 or 1uM) or 0.01% DMSO as vehicle was added for additional 24 h. HCL cell death was analyzed by flow cytometry as described in the legend of **Figure 1**. Shown are the results for the entire cohort (n = 8). **(C)** PBMC from HCL patients were co-cultured with HS-5 stromal cells for 48 h and labeled with anti-CD19 and anti-CD86. Shown are representative dot plots (left) and mean values (right) for n = 3 (two-tailed paired *t-*test). **(D)** PBMCs from HCL patients were co-cultured with HS-5 for 48 h. Then venetoclax (0.1 or 1uM) or 0.01% DMSO as vehicle was added for additional 24 h, and HCL cell death was analyzed as described above. **(E)** PBMCs from HCL patients were incubated with medium alone (Ctrl) or in the presence of PAM3 (300 ng/ml) or CpG (5 μM). At 48 h, cells were labeled with anti-CD19 and anti-CD86. Shown are representative dot plots (left) and mean values (right) for n = 7. **(F)** PBMCs from HCL patients were incubated with medium alone (Ctrl) or in the presence of PAM3 (300 ng/ml) or CpG (5 μM) for 24 h. Then venetoclax (0.1 or 1uM) or 0.01% DMSO as vehicle was added for additional 24h and HCL cell death was analyzed as described above. Shown are the results for the cohort studied (n=7). For all the group graphics, each symbol represents a single patient and paired one-way ANOVA followed by multiple comparisons were performed unless otherwise indicated (*p < 0.05, **p < 0.01, ***p < 0.001, ****p < 0.0001, ns, not significant).

We next determined whether activation of HCL cells through signals from a supportive microenvironment could impair venetoclax-induced cell death. Since CD40–CD40L triggering plus IL-4 represents a known activator of HCL cells (27), we first tested if *α*CD3 stimulation could mimic such phenomenon. After 24 h of stimulation, HCL cells upregulated CD69 due to the presence of autologous activated T cells by CD3 engagement (**Supplementary Figure S3A**), and after 48 h of culture, a significant increase in CD86 positive HCL cells was observed (**Figure 2A**). Upon stimulation, HCL cells were highly resistant to venetoclax-induced death at both doses evaluated (**Figure 2B**). This was also the case when HCL-derived PBMC samples were co-cultured with the stromal cell line HS-5. As shown in **Figures 2C, D** and in **Supplementary Figure S3B**, HCL co-cultured with the HS-5 cells increased their size and granularity, upregulated CD86, and showed diminished values of Annexin V positivity when treated with venetoclax at 0.1 and 1 µM.

Finally, we evaluated the role of Toll-Like Receptor (TLR) engagement in the activation of primary HCL samples and studied their potential to affect venetoclax-induced cell death of tumor cells. For such aim, we incubated patient-derived PBMCs with CpG (5 µM) or PAM3 (300 ng/µl), ligands for TLR9 and TLR2, respectively. A significant increase in the expression of CD86 in HCL cells was observed after 48 h of stimulation with PAM3 (**Figure 2E**), whereas CpG did not induce statistically significant effects of activation markers in our cohort (**Supplementary Figure S3A**). **Figure 2F** shows the levels of death induced by venetoclax on HCL exposed to PAM3 or CpG for 24 h. A consistent decrease of Annexin V+ HCL cells for both stimuli was detectable when venetoclax was used at 0.1 or 1 µM, suggesting a protective role for TLR9 and TLR2 signaling in venetoclax-induced cell death of HCL cells.

## Discussion

In the present work, we focused on studying the effect of venetoclax in HCL *in vitro* and chose to work with patient-derived samples. We forwarded into this approach because previous reports using HCL cell lines detected absence of key hallmarks of this disease such as BRAF-V600E mutation (28, 29) and differences in sensitivity to drugs such as Ibrutinib as compared to their effect on primary HCL samples (30). Additionally, inherent resistance to Bcl-2 inhibition by venetoclax has been previously reported in tumor-derived human B cell lines (31). In our series of eight HCLs, two patients belong to the variable HCL group and showed similar values of venetoclax-induced cell death, of activation by the three stimuli tested, and of their protection capacity towards cell death induction, compared to the six classic HCL patients enrolled. The same occurred with the only patient that belongs to the VH4-34 molecular variant group of HCL, sample where the parameters evaluated were comparable to the remaining three in which we could identify the immunoglobulin (IG) variable heavy chain gene of use. Similarly, venetoclax-induced cell death was achieved independently of the IG light chain usage, the percentage of circulating HCL cells, white blood cell or platelet counts, sex, or age of the patient.

The main limitation of our study is the low number of samples analyzed. Testing primary HCL samples remains a difficult task mainly due to the low frequency of the disease. Besides being a rare neoplasia, as most patients develop pancytopenias, a limited amount of leukemic cells is usually obtained from peripheral blood, and extraction during bone marrow biopsies faces the additional complication of fibrosis. Furthermore, cladribine-treated patients remain with undetectable circulating leukemic cells for years. For us, all these characteristics resulted in little available material to increase the size of the cohort of study, vary concentrations and exposures to the drug or to further into the mechanisms of action and deepen into the role of Bcl-2 inhibition in this leukemia.

## Conclusion

We have observed that venetoclax is able to induce a clear pro-apoptotic effect in primary HCL cells. This effect does not appear to operate differently when studying samples derived from the classic, variant, or VH4-34 forms of the disease. HCL cells can be activated by co-culture with stromal cells and by TLR triggering, as already shown for T-cell mediated stimulation. Activation of HCL leads to resistance to venetoclax-induced cell death. The signals provided by the accessory cells that compose the HCL microenvironment in the bone marrow or spleen of patients could mimic such activation *in vivo* in the human disease. We propose that venetoclax could be considered for HCL therapy, and that combinatory strategies with drugs that target the microenvironment may improve its efficacy.

## Data Availability Statement

The raw data supporting the conclusions of this article will be made available by the authors, without undue reservation.

## Ethics Statement

Written informed consent was obtained from the individual(s) for the publication of any potentially identifiable images or data included in this article.

## Author Contributions

PM, MG, MB, and RG designed the research. PM and MG wrote the manuscript. AV, PM, MG, AC, and EG performed experiments and analyzed data. EM and JP collaborated in the design and data presentation. RB, HF, GC, MC, J-HF, and GB provided patient samples and advice and contributed in the interpretation of the data. All authors contributed to the article and approved the submitted version.

## Funding

This work was supported by grants from the Agencia Nacional de Promoción Científica y Tecnológica (PICT 0290-2015), CONICET, Argentina; from FNR Luxembourg (PRIDE15/10675146/CANBIO and INTER/DFG/16/11509946), and from FNRS “Télévie” (7.8506.19).

## Conflict of Interest

RB receives payment from Microsules and Varifarma. GC receives payment for lectures from Janssen. RC receives payment for lectures from AtraZeneca.

The remaining authors declare that the research was conducted in the absence of any commercial or financial relationships that could be construed as a potential conflict of interest.

## Publisher’s Note

All claims expressed in this article are solely those of the authors and do not necessarily represent those of their affiliated organizations, or those of the publisher, the editors and the reviewers. Any product that may be evaluated in this article, or claim that may be made by its manufacturer, is not guaranteed or endorsed by the publisher.
