# Supplementary material for: In Vitro Sensitivity to Venetoclax and Microenvironment Protection in Hairy Cell Leukemia
Source: Front Oncol. 2021 Jul 26;11:598319. doi: 10.3389/fonc.2021.598319 (PMC8350736; doi:10.3389/fonc.2021.598319)
Supplement: Supplementary file 1 [file DataSheet_1.zip › Supplementary Table 1.pptx]

## Slide 1
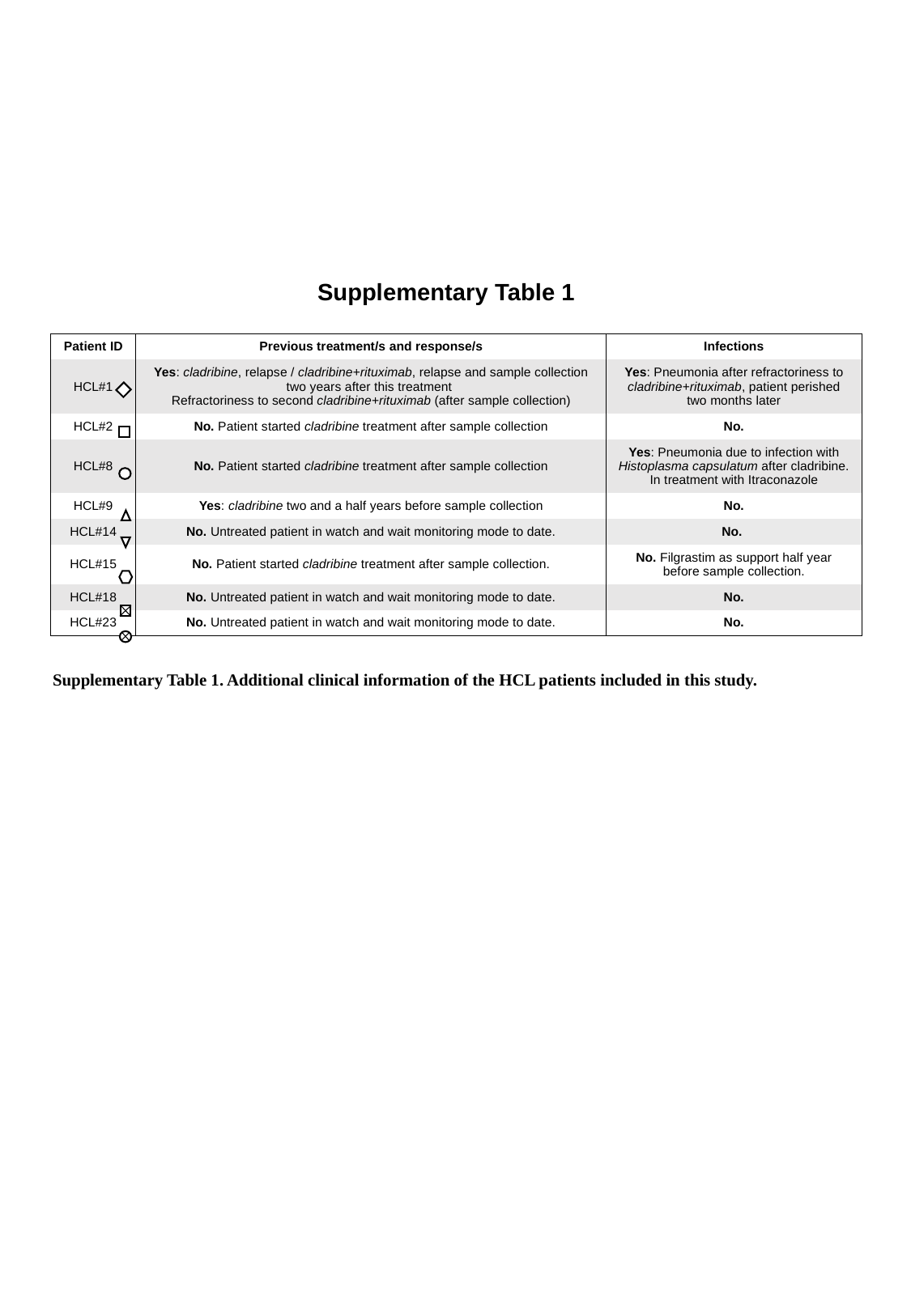

Supplementary Table 1
| Patient ID | Previous treatment/s and response/s | Infections |
| --- | --- | --- |
| HCL#1 | Yes: cladribine, relapse / cladribine+rituximab, relapse and sample collection two years after this treatment Refractoriness to second cladribine+rituximab (after sample collection) | Yes: Pneumonia after refractoriness to cladribine+rituximab, patient perished two months later |
| HCL#2 | No. Patient started cladribine treatment after sample collection | No. |
| HCL#8 | No. Patient started cladribine treatment after sample collection | Yes: Pneumonia due to infection with Histoplasma capsulatum after cladribine. In treatment with Itraconazole |
| HCL#9 | Yes: cladribine two and a half years before sample collection | No. |
| HCL#14 | No. Untreated patient in watch and wait monitoring mode to date. | No. |
| HCL#15 | No. Patient started cladribine treatment after sample collection. | No. Filgrastim as support half year before sample collection. |
| HCL#18 | No. Untreated patient in watch and wait monitoring mode to date. | No. |
| HCL#23 | No. Untreated patient in watch and wait monitoring mode to date. | No. |
Supplementary Table 1. Additional clinical information of the HCL patients included in this study.
